# Visual estimation of blood loss by UK pre-hospital clinicians: an observational study

**DOI:** 10.29045/14784726.2018.06.3.1.16

**Published:** 2018-06-01

**Authors:** Michael Liam Townend, Sonia Byers

**Affiliations:** Northumbria Healthcare NHS Foundation Trust; Leica Biosystems, Newcastle University

**Keywords:** continuity of patient care, emergency medical services, haemorrhage

## Abstract

**Aim:**

To assess the accuracy of visual estimation of external blood loss by UK pre-hospital clinicians and to comment on its value during handover.

**Methods:**

A sample of 104 pre-hospital clinicians were shown eight staged scenarios showing varying amounts of blood loss and asked to estimate the amount of blood loss depicted. Participants included a range of pre-hospital clinicians from both NHS ambulance trusts and Helicopter Emergency Medical Services.

**Results:**

A wide distribution of estimates and therefore percentage error was observed in our study. Pre-hospital clinicians are inaccurate when estimating external blood loss at scene, regardless of training and skill level.

**Conclusion:**

Visual estimation of blood loss is too inaccurate to be considered clinically worthwhile. Greater research focus is needed to investigate and validate better measures of blood loss that can be utilised in the pre-hospital and emergency medicine environment. Until evidence-based methods of estimation can be implemented, this information should not be included in hospital handovers.

## Introduction

Trauma is the leading cause of mortality in under 40-year-olds across the world, with haemorrhage accounting for 30–40% of trauma deaths in the United States (Kauvar, Lefering, & Wade, 2006).

In guidelines recently published by the National Institute for Health and Care Excellence (2016) it is estimated that there are approximately 15,000 deaths due to accidents per year in the UK. The guidelines state that uncontrolled haemorrhage is one of the leading causes of death after injury and that early detection of haemorrhagic shock, specifically in patients requiring massive transfusion, could substantially improve outcomes.

In a recent publication, Barnard, Yates, Edwards, and Fragoso-Iniguez (2017) interrogated the Trauma Audit and Research Network database to examine the aetiology of traumatic cardiac arrest in England and Wales. Their results suggested that 35.6% of their 705-included patient population suffered cardiac arrest secondary to haemorrhage. In this study, haemorrhage was the highest single cause of traumatic cardiac arrest, second only to the combination of traumatic brain injury and haemorrhage (Barnard et al., 2017).

In the pre-hospital setting, recognition of hypovolaemic shock is made primarily using two parameters – visual estimation of external blood loss and the patient’s vital signs in accordance with the widely taught and accepted Advanced Trauma Life Support (ATLS) programme (American College of Surgeons Trauma Committee, 2008). Studies in both the UK and Germany have questioned the validity of these ATLS guidelines, particularly in reference to the relationship between haemodynamic parameters (pulse, blood pressure and respiratory rate) and estimated blood loss (Guly et al., 2010; Mutschler et al., 2012). Earlier detection of hypovolaemia therefore requires greater attention towards improving the methods of estimating blood loss. One such method is visually estimating the volume of blood that a patient has lost by carefully observing the scene. Studies in America and Australia have shown that this method is inaccurate but can be improved with training (Buckland & Homer, 2007; Frank et al., 2010; Patton, Funk, McErlean, & Bartfield, 2001; Tall, Wise, Grove, & Wilkinson, 2003). A detailed literature search by the author has shown that no pre-hospital studies have been conducted in the UK.

Clinicians within emergency departments rely on information provided by pre-hospital clinicians to inform and direct initial patient care. The authors contacted the research and development leads for all 10 English ambulance services and found that the majority (nine) of trusts’ patient report forms (PRFs) contained a section for the clinician to provide an estimate of blood loss. As a result, visually estimated on-scene blood loss is often included in the handover of information, potentially misleading hospital clinicians with regards to extent of injury and inadvertently influencing their treatment decisions. It is therefore important to establish the accuracy of visual estimation of blood loss to determine whether this information should be provided by pre-hospital clinicians at the point of patient handover.

The aim of this study was to create and utilise a multimedia tool in order to assess the accuracy of visual estimation of external blood loss by a sample of UK pre-hospital clinicians. The authors also planned to use the results to comment on the clinical value of providing estimated blood loss to the receiving clinicians during hospital handover.

## Methods

### Artificial blood

A large volume of artificial blood was made following a recipe used in an Australian study (Buckland and Homer, 2007) examining estimation of blood loss among obstetric clinicians. Care was taken to ensure that the artificial blood was as close to the same colour and consistency of real blood as possible. The diameters of the three blood pools were compared to the expected surface areas stated in the Institute of Health and Care Development (2011, section 13.1) *Basic Training Manual* and were found to be comparable. The IHCD manual was chosen as it was felt by the authors that any training in estimation of blood loss received by the participants was most likely to have been based on this resource. When analysing the response forms the authors found that two thirds of the paramedic participants were IHCD trained.

The authors were keen to use real blood but, as discussed in the limitation section, this would have significantly restricted the scope and practicality of this study.

### Scenarios

The authors created eight scenarios incorporating simulated blood loss with artificial blood being added to the scenario using syringes and measuring jugs to allow accurate measurement. The scenarios are shown in Table 1. Blood volumes and the blood flow depicted in the videos were felt by the authors to be proportionate to the mechanism of injury.

**Table 1.** Scenarios.

Scenario	Surface	Actual blood loss (ml)
1. Blood pool	White linoleum flooring, non-porous	250
2. Blood pool	Light coloured carpet	250
3. Blood pool	Dark coloured deep pile carpet	250
4. Epistaxis	Several 2-ply tissues in sink, plug in place	32.5
5. Self-inflicted wrist injury	Dark linoleum, clothing and wound dressing	50
6. Head injury	Tarmac and patient hair	200
7. Stabbing	Tarmac and clothing	300
8. Leg injury	Grass and clothing	1400

The scenarios were photographed from multiple angles and a short video clip of the scene was recorded. The camera used was a Panasonic DMC-FZ45, 14MP, 24× optical zoom and the video clips were shot in 1080p High Definition. Scenes 1 to 5 were conducted indoors with artificial and natural lighting, while scenes 6 to 8 were conducted outdoors. Images from each scene were combined into a film reel using iMovie ’11® (Apple Inc.). Still images were used for scenes 1 to 5, while video clips were added to scenes 6 to 8 as they were felt by the authors to offer additional information to the viewer.

The total viewing time for each scenario was deliberately kept short (10 seconds for scenes 1 to 5 and 30 seconds for scenes 6 to 8) to reduce the amount of time participants spent viewing the whole video and thus minimise any inconvenience to participants.

Examples of two scenarios are shown in Figure 1. The whole video can be viewed at https://www.youtube.com/watch?v=2QDwti7eugI.

**Figure:**
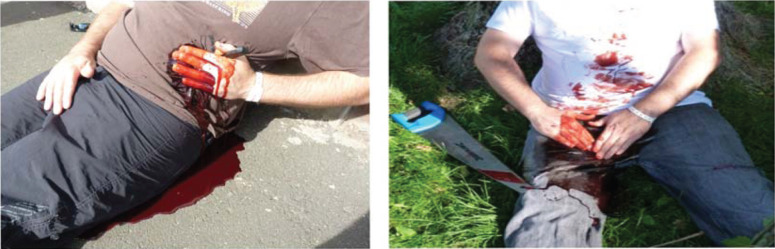
Figure 1. Example stills from the scenarios.

### Participants

The study population was comprised of a convenience sample of pre-hospital clinicians from an English NHS ambulance service and one Helicopter Emergency Medical Service (HEMS). Participants were approached either outside of A&E during an operational shift or during annual mandatory training. HEMS personnel were approached during clinical governance training days. Permission was gained from both organisations to approach and recruit their members of staff during the course of the study. Permission was also gained from the local hospital trust to recruit ambulance crews outside of their emergency department.

Following a brief explanation of the aims of the study, they were presented with a participant information leaflet; adequate time was allowed for them to read the information and to have any questions answered, after which consent was recorded on a participant consent form, ensuring that they understood that participation was voluntary and that they were free to decline to take part at any point. All participants were given contact details in the event that they withdrew consent after the data had been collected. Eighteen ambulance service individuals declined to take part at the time they were approached during an operational shift. No one declined to participate when approached during a training or clinical governance day. No participants withdrew their consent at a later date.

Once the consent form had been completed, participants were invited to view the video containing all eight simulated scenarios on a 13-inch High Definition laptop screen. They were asked to view the entire video once only, estimate the volume of blood loss within each scene in millilitres and record their answer on the response form. Viewing the video was restricted to one time only to standardise the intervention across the sample group and to minimise inconvenience to the participant. The authors also felt that this helped to reflect the time pressure experienced by pre-hospital clinicians when dealing with injured patients on scene. They were asked to provide a single estimate, rather than a range, and were not permitted to change their answer afterwards. In addition, participants provided demographic information relevant to gender, role, pre-hospital care experience and whether or not they had received formal training on the visual estimation of blood loss.

### Analysis

Data from the response forms were transferred to Microsoft Excel. Absolute and percentage error (response volume – actual volume/actual volume × 100) were calculated for each response. Descriptive statistics for both absolute error and % error (median, mean (SD), upper and lower quartile and minimum and maximum values) were then calculated. This process was repeated for all sub groups to allow the authors to study the impact of role, pre-hospital care experience, previous formal training and gender on the accuracy of estimation.

## Results

One hundred and four pre-hospital clinicians participated in the study, providing 832 estimates of blood loss (Table 2). The majority of participants were paramedics, with 10 working as full time HEMS team members and 61 being based within an NHS ambulance service. Fourteen HEMS doctors and one HEMS nurse practitioner were also included in the study. The majority (75%) of participants were male and 85.5% of participants reported greater than five years’ pre-hospital experience. Only 15 participants reported having received formal teaching in the visual estimation of blood loss and all of these quoted the IHCD manual as the basis of their training.

**Table 2.** Demographic characteristics of participants.

Characteristics		N (%)
Total participants	104	(100)
Role:		
Paramedic	71	(68.3)
Doctor	14	(13.5)
Emergency care support worker	13	(12.5)
IHCD technician	5	(4.8)
Advanced nurse practitioner	1	(1)
Sex:		
Male	78	(75)
Female	26	(25)
Pre-hospital experience (years):		
< 1	5	(4.8)
1–2	2	(1.9)
3–4	8	(7.7)
5–10	33	(31.7)
> 10	56	(53.8)
Received training:		
Yes	15	(14.4)
No	89	(85.6)

The greatest frequency of overestimation was seen in scene 5 (50 ml), with 90 participants overestimating and a median percentage error of 200%. There was also significant overestimation in scene 7 (300 ml), where 82 participants overestimated, with a median percentage error of 125%.

The greatest degree of underestimations was seen in scene 8, which contained the largest volume of blood loss of 1400 ml. Ninety-four participants underestimated the blood volume, with a median percentage error of −64.3%. Participants also underestimated scene 3 (250 ml pool on dark carpet), with 75 participants overestimating and a median percentage error of −40%.

The first three scenarios contained the same volume of blood lost on three different surfaces. There were similar numbers of estimation within 20% of the actual volume but more participants overestimated the volume on the linoleum and light carpet. Only four participants recognised that scenes 1 to 3 contained the same volume, though none estimated accurately.

Sub group analysis showed that role, gender, experience and previous training on estimation of blood loss had no discernible effect on the participants’ accuracy of estimation. No perceived difference was noted between HEMS based and ambulance service based paramedics.

## Discussion

The results observed within our study match those seen in studies from the United States and Australia (Beer, Duvvi, Webb, & Tandon, 2005; Moscati et al., 1999; Patton et al., 2001; Tall et al., 2003). As a group, pre-hospital care clinicians are not able to accurately estimate external blood on-scene. There is a wide variation in both estimated volumes and therefore percentage error. There appears to be no correlation between roles, experience, gender or previous training with regards to the accuracy of estimation, which again supports previous findings. It would therefore be appropriate to comment that visual estimation of blood loss is too inaccurate to be of any significant clinical use.

Although it appears that the medians of the estimates provided by participants are close to the actual amount, very few participants provided consistently accurate estimates (Table 3). Of the participants, 90% did not provide more than three estimates within 20% of the actual blood volume depicted in the scenarios. Only one participant estimated five amounts within 20%; no one had more than five estimates within 20% of the correct amounts. The 20% margin was used in our study only in order to assess participants’ consistency with regards to estimation across all eight scenarios and is not meant to confer ‘accuracy’ of estimation.

**Table 3.** Blood loss estimates stratified by scenario.

Scene	Actual blood loss (ml)	Median absolute error (IQR, ml)	Median percentage error (IQR, %)	Overestimated blood loss (n)	Underestimated blood loss (n)	Accurate(n)	Estimation within 20% of actual (n)
1	250	−50	(−150–50)	−20	(−60–20)	30	61	13	34
2	250	0	(−137.5–150)	0	(−55–60)	45	51	8	35
3	250	−100	(−183.75–0)	−40	(−73.5–0)	16	75	13	32
4	32.5	17.5	(−12.5–67.5)	53.8	(−38.5–207.7)	69	35	0	9
5	50	100	(42.5–237.5)	200	(85–475)	90	9	5	10
6	200	50	(−50–137.5)	25	(−25–68.75)	57	32	15	17
7	300	375	(50–700)	125	(16.7–233.3)	82	15	7	20
8	1400	−900	(−1050–−425)	−64.3	(−75–−30.4)	10	94	0	6

Overestimation of smaller volumes of blood loss as seen in the majority of our scenes may be of less clinical consequence, but of greater concern was the underestimation witnessed within scene 8, for which the blood volume was 1400 ml (approx. 40% of total blood volume) (Figure 2). Failure to recognise this degree of blood loss could have adverse consequences on patient management and ultimately patient outcome. The expectation according to ATLS guidelines is that a patient suffering from acute severe blood loss would demonstrate sufficient haemodynamic compromise (increased heart rate, lowered systolic blood pressure and increased respiratory rate) as to alert pre-hospital clinicians to the patient’s condition, irrespective of witnessed blood loss (American College of Surgeons Trauma Committee, 2008). However, the work by Guly et al. (2011) suggests that many patients with severe blood loss (> 40% total blood volume) do not demonstrate these physiological changes. Indeed, the values obtained within their study showed that the median values for heart rate, blood pressure and respiratory rate fall outside the parameters that would be expected if following ATLS classification. Many of these patients would appear to be more physiologically well than their blood loss would suggest. This could result in delayed detection of hypovolaemia, less assertive resuscitation and fewer pre-alerts to the receiving hospital. In terms of major trauma patients, there is likely to be little impact from the results of this study. While there are some minor regional variations to the major trauma triage tool, the focus of decision-making is based on mechanism of injury, injury patterns and on the patient’s physiology.

**Figure:**
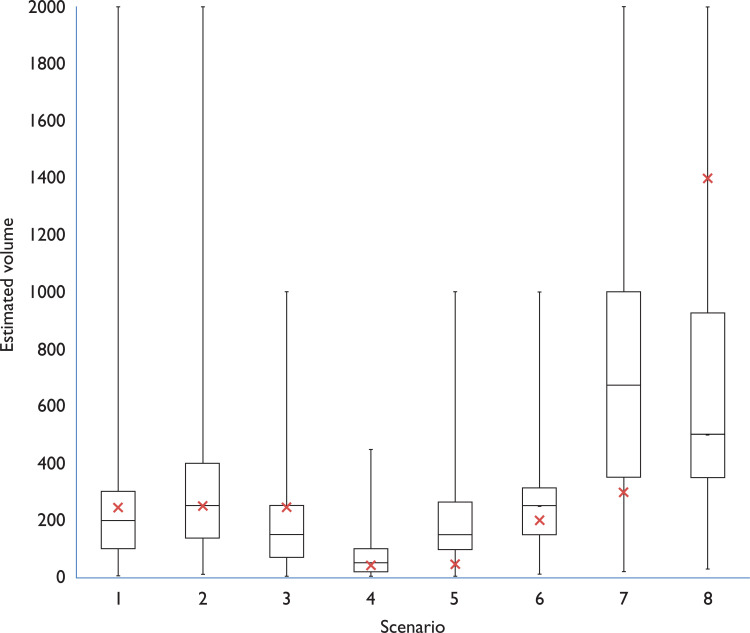
Figure 2. Distribution of estimates by scenario.

Red crosses show actual amounts depicted in scenarios.

Overestimation, as seen in scenes 5 and 7, may have resulted from the implied violence in the scene (self-harm and stabbing), causing the participants to predict a higher injury severity and therefore blood loss. Pronounced underestimation, as seen in scene 8, may have been due to the high absorbency of the clothing and surfaces, misleading the participants with regards to blood volume. These scenes demonstrate the diverse environment in which pre-hospital clinicians operate and therefore the challenges that are presenting when attempting to estimate blood loss.

Only four participants correctly guessed that the amounts in the first three scenarios were identical, although none of these guessed the correct amount. The scenes showing 250 ml blood pools on linoleum and short pile carpet showed similar distributions of estimate while the scene using deep pile, darker carpet showed a greater degree of underestimation. This suggests that any future work to improve estimation should include the effect that different surfaces may have on saturation and distribution of blood.

It is important for the authors to comment on the implications of these results to pre-hospital practice in the UK in relation to management and transfer of trauma patients.

A relatively recent addition to the paramedic arsenal when managing trauma patients is the antifibrinolytic agent, tranexamic acid. The indications for tranexamic acid as stated in the Joint Royal College Ambulance Liaison Committee (Brown, Kumar, Millins, & Mark, 2016) guidelines are for patients with time critical injury where significant internal or external haemorrhage is suspected and in patients suffering from post-partum haemorrhage. A Cochrane review into the use of tranexamic acid in trauma patients found that its use conferred a reduction in mortality in bleeding trauma patients without increasing the risk of adverse events (Ker, Roberts, Shakur, & Coates, 2015). It is the authors’ experience that the decision to administer tranexamic acid in a trauma patient is generally influenced by a combination of the mechanism of injury, suspected injury pattern and the patient’s physiological parameters, and not by visually estimated blood loss. Therefore, it is not anticipated that the results of this study will have any significant impact on pre-hospital tranexamic acid use.

A combination of physiological parameters coupled with more accurate reflection of the blood loss may help to improve detection of hypovolaemic shock. It is clear that further work must be conducted to address this evidence gap in order to ensure that trauma patients in the UK are receiving the highest level of care and achieving the best possible outcomes.

There is a paucity of research into training pre-hospital in visual estimation of external blood loss; however, this topic has been examined extensively within the obstetric environment. A 2006 study by Bose, Regan, and Paterson-Brown (2006) looked at the accuracy of visually estimated blood loss by a range of clinicians in the context of post-partum haemorrhage. They created 12 clinical OSCE stations containing known volumes of real blood, which they asked participants to rotate through and estimate the blood loss witnessed at each station. As is the case with this study, they found a wide range of observations that did not follow a normal distribution. Five of their 12 stations were significantly underestimated and no station was overestimated. The authors then produced a simple pictorial guide to be placed in clinical areas to aid and improve clinicians’ estimation (Bose et al., 2006). In a similar study, Zuckerwise, Pettker, Illuzzi, Raab, & Lipkind (2014) assessed the impact of using a pictorial guide on participants’ accuracy of estimating blood loss. They found that the use of a simple, reproducible visual guide significantly improved visual estimation of blood in four out of six scenarios (Zuckerwise et al., 2014).

These two studies show promise for improving the accuracy of estimation of blood loss by pre-hospital clinicians. However, these studies were performed in a controlled hospital ward environment, which does not reflect the pre-hospital setting where environmental factors such as surface type, lighting and personnel have a much greater bearing. The first three scenarios in our study showed 250 ml blood pools on different surfaces. Only four participants recognised that the pools were the same volume, and none estimated correctly. This suggests that any future work to develop training aids to improve estimation needs to investigate and address the effect that different surfaces may have on saturation and distribution of blood.

## Limitations

Although the sample size was reasonable, in the context of an opportunistic sampling strategy, and was thought to represent a cross section of pre-hospital clinicians, more data would increase the confidence in the conclusions and would also allow more accurate sub-analysis of the different skill levels.

The sample population consisted primarily of para-medics (68%), resulting in smaller groups representing other roles, making accurate subgroup analysis difficult. However, the vast majority of UK pre-hospital care is currently delivered by paramedics, so the sample group is thought to be representative of the environment. It would be useful, if the study were repeated, to increase the numbers of HEMS crews that were recruited to enable better comparison between HEMS and non-HEMS paramedics.

The authors explored the possibility of using real blood in order to make the simulation as high fidelity as possible. NHS Blood and Transplant (NHSBT) can provide non-clinical issue blood products for research purposes, but there are strict regulations around biohazard controls and disposal. Given that some of the scenarios were to be filmed outdoors in public areas and involved applying the blood to the skin of ‘casualties’, the decision was made to use artificial blood.

The blood used in the scenarios was created using food products. While this serves to minimise both costs and the risk of blood-borne infection, it is difficult to accurately produce the same viscosity and flow characteristics of real blood. There is also no clotting effect within our samples, which may have influenced the results seen, though research blood provided by NHSBT is treated to prevent coagulation so would have presented a similar problem.

Participants were only given a short time in which to view each image and were only permitted to view the whole video once. While this served to reduce inter-subject variation and any potential inconvenience to the participants, it may have actually hindered their estimation attempts. Clearly these scenarios do not take into account the inevitable time and environmental pressures placed on clinicians when attending patients out of hospital. They also do not take into account differing environmental conditions such as light, weather or noise.

The gold standard protocol for this project would have been to create the scenarios and have the study participants view them in situ, as they would during their everyday roles. However, this was felt to be logistically challenging and would limit the number of participants who could be enrolled in the study.

## Conclusion

The results of this study suggest that UK pre-hospital clinicians are generally unable to accurately visually estimate external blood loss. During handover, clinicians should be mindful that estimates could be inaccurate by a significant margin. It may be more useful to include a qualitative statement with regards to blood loss alongside the patient’s physiological parameters during hospital handover. Greater focus needs to be placed on investigating and validating better methods for estimating blood loss that can be applied practically in the pre-hospital and emergency medicine environments.

## Acknowledgements

The authors would like to thank Mr James Hitch for his time and assistance with photography and Frank’s The Flooring Centre for providing free carpet and linoleum samples. With special thanks to my tutor Dr Anthony Hudson and to Ms Emily McWhirter (KSS HEMS).

## Conflict of interest

None declared.

## Ethics

All participants provided informed consent, evidenced by signing a written consent form. Given the nature of this study, ethical approval was not required. North East Ambulance Service NHS Foundation Trust and Kent, Surrey and Sussex Air Ambulance Trust provided permission for their staff to be approached for data collection.

## Funding

None.
